# Farnesylation of the Transducin G Protein Gamma Subunit Is a Prerequisite for Its Ciliary Targeting in Rod Photoreceptors

**DOI:** 10.3389/fnmol.2018.00016

**Published:** 2018-01-23

**Authors:** Celine Brooks, Joseph Murphy, Marycharmain Belcastro, Daniel Heller, Saravanan Kolandaivelu, Oleg Kisselev, Maxim Sokolov

**Affiliations:** ^1^Department of Ophthalmology, West Virginia University, Morgantown, WV, United States; ^2^Department of Ophthalmology, Saint Louis University, St. Louis, MO, United States; ^3^Department of Biochemistry, West Virginia University, Morgantown, WV, United States

**Keywords:** retina, rod photoreceptor, heterotrimeric G protein, farnesylation, protein trafficking, cilium

## Abstract

Primary cilia are microtubule-based organelles, which protrude from the plasma membrane and receive a wide range of extracellular signals. Various cilia use G protein-coupled receptors (GPCRs) for the detection of these signals. For instance, vertebrate rod photoreceptors use their cilia (also called outer segments) as antennae detecting photons by GPCR rhodopsin. Rhodopsin recognizes incoming light and activates its G protein, transducin, which is composed of three subunits α, β, and γ. Similar to all G protein γ subunits, the transducin Gγ_1_ subunit undergoes C-terminal prenylation resulting in the addition of an isoprenoid farnesyl; however, the significance of this posttranslational modification is unclear. To study the role of the farnesyl group, we genetically introduced a mutant Gγ_1_ that lacked the prenylation site into the retinal photoreceptors of mice. The biochemical and physiological analyses of these mice revealed that mutant Gγ_1_ dimerizes with the endogenous transducin Gβ_1_ subunit and that the resulting Gβγ dimers display reduced hydrophobicity. Although mutant Gβγ dimers could form a heterotrimeric G protein, they could not mediate phototransduction. This deficiency was due to a strong exclusion of non-farnesylated Gβγ complexes from the cilia (rod outer segments). Our results provide the first evidence that farnesylation is required for trafficking of G-protein βγ subunits to the cilium of rod photoreceptors.

## Introduction

Ciliary signaling is commonly mediated by G protein-coupled receptors (GPCRs), which are integral membrane proteins (Schou et al., 2015). Their coupled heterotrimeric G proteins are soluble proteins attached to the lipid bilayer through posttranslational lipid modification. The difference in their lipid bilayer interaction underlies distinct cilia targeting mechanisms for GPCRs and G proteins, which is exemplified in retinal rod photoreceptors of vertebrates.

Rods use their cilia, called outer segments, as antennae specialized for photon detection. The outer segment is packed with flat lamellar membranes, or discs, containing photosensory GPCR, rhodopsin, coupled to heterotrimeric G protein, transducin (Pearring et al., 2013). When activated by light, rhodopsin signals transducin triggering a chain of signaling events leading to rod hyperpolarization and synaptic response. It is generally accepted that rhodopsin is added to the disc membrane through a distinct trafficking pathway regulated by Arf and Rab GTPases (Wang and Deretic, 2014). Nascent discs originate from lamellae, which grow outward from the outer segment and either evaginate or invaginate from the ciliary plasma membrane (Steinberg et al., 1980; Burgoyne et al., 2015; Ding et al., 2015; Volland et al., 2015). Fusion of the leading edges of adjacent lamellae results in formation of discrete discs, which are no longer continuous with the ciliary plasma membrane. Then the concentration of any integral membrane protein present on the disc, including rhodopsin, does not change. At the same time, the amount of transducin in the rod outer segment is variable. In resting rods, transducin accumulates in outer segment discs, which maximizes the rate of transducin activation by rhodopsin, hence, the sensitivity of rod responses. However, during sustained exposure to bright ambient light which saturates rods, a major fraction of transducin undergoes translocation from the rod outer segment to the cell body. This results in a drastic reduction in the concentration of transducin in the outer segment (Brann and Cohen, 1987; Philp et al., 1987; Whelan and McGinnis, 1988; Sokolov et al., 2002; Nair et al., 2005; Lobanova et al., 2007; Rosenzweig et al., 2007). This response is reversed in the dark, when the displaced transducin returns to the outer segment and re-populates discs (Sokolov et al., 2002; Belcastro et al., 2012). Previous research indicates that transducin exits outer segments by diffusion, after losing its affinity to disc membrane, which highlights the central role of lipid groups in this process (reviewed in Calvert et al., 2006; Artemyev, 2008; Slepak and Hurley, 2008; Pearring et al., 2013).

Rod transducin is a prototypical heterotrimeric G protein comprised of α subunit and a complex of tightly associated β and γ subunits. The α and γ subunits are lipid-modified, which is required for their membrane association and protein interaction (Wedegaertner et al., 1995). Similar to other G-protein γ subunits, rod transducin γ (Gγ_1_) carries a covalently-attached isoprenoid, farnesyl (Fukada et al., 1990). The prenylation reaction, the addition of an isoprenoid group either farnesol or geranylgeraniol, is directed at cysteine residue 71 within a carboxyl-terminal CAAX box (C^71^VIS) displayed by the nascent Gγ_1_ (Lai et al., 1990). The isoprenoid attachment is followed by the removal of the last three amino acids (VIS) and methylation of the carboxyl group of the new c-terminus (Higgins and Casey, 1994). Thus, mature Gγ_1_ is three amino acid residues shorter than its precursor polypeptide and its c-terminal C^71^ is farnesylated and methylated. While the prenylation of the G-protein γ subunits is not required for the assembly of the βγ dimer, it is indispensable for the membrane targeting of Gβγ (Simonds et al., 1991). In addition to membrane targeting, prenylation enhances the binding of Gβγ to G-protein α subunits (Iñiguez-Lluhi et al., 1992). Currently there is a lack of *in vivo* experiments, assessing why farnesylation of Gγ is important for visual function. Some evidence suggests that posttranslational lipid modification controls transducin compartmentalization in retinal photoreceptors. For example, replacing the farnesyl group of Gγ_1_ with the more hydrophobic isoprenoid group, geranylgeranyl renders the transducin Gβ_1_γ_1_ dimer incapable of undergoing light-driven translocation from rod outer segments (Kassai et al., 2005). In *Drosophila*, farnesylation of transducin Gγe was critical for the formation of membrane-associated Gαβγ heterotrimer competent to interact with its GPCR (Schillo et al., 2004). However, it is not known if this role is also true for vertebrates or if there are other roles. Our data provide the first evidence that farnesylation of transducin Gγ_1_ is also necessary for targeting of the Gβ_1_γ_1_ dimer to the sensory cilium of vertebrate rod photoreceptors *in vivo*.

## Materials and Methods

### Generation of ^HA^Gγ_1_ and ^HA^Gγ_1_C71S Transgenic Mice

A transgene expressing the γ subunit of rod-specific transducin (protein: Gγ_1_, gene: *Gngt1*) was synthetized by Integrated DNA Technologies (IDT, Coralville, IA, USA). This construct designated as ^HA^Gγ_1_ contained HA epitope tag, LMA linker, mouse *Gngt1* coding sequence, and a 4.4 kb mouse rhodopsin promoter (Lem et al., 1991). The cysteine-to-serine substitution at position 71, resulting in ^HA^Gγ_1_C71S mutant lacking the prenylation site, was introduced by a PCR-based strategy and the QuikChange Lightning Site-Directed Mutagenesis Kit (Agilent Technologies, Santa Clara, CA, USA) with the following primers: forward primer 5′-AAG GAA CTC AAA GGA GGC TCT GTG ATT TCA TAG TAG G and reverse primer 5′-CCT ACT ATG AAA TCA CAG AGC CTC CTT TGA GTT CCT T (with the underlined base indicating the change from wild type (WT) sequence). The integrity of both constructs was confirmed by sequence analysis. Then they were purified and injected into the pronuclei of zygotes from superovulated FVB females at the WVU Transgenic Animal Core Facility. Transgene integration was determined for both groups by PCR genotyping of tail DNA using the following primers: forward primer 5′-TAC CCA TAC GAT GTT CCA GAT TAC GCT and reverse primer 5′- TCA CAC AGC CTC CTT TGA GTT CCT. The colonies were established by crossing transgenic ^HA^Gγ_1_^+/−^ and ^HA^Gγ_1_C71S^+/–^ heterozygotes with WT partners of 129-E background (Charles River). To move both transgenes to Gγ_1_-null background, ^HA^Gγ_1_^+/−^ and ^HA^Gγ_1_C71S^+/–^ heterozygotes were subjected to several round of crossing with Gγ_1_^−/−^ mice (Kolesnikov et al., 2011) to obtain ^HA^Gγ_1_^+/−^ ; Gγ_1_^−/−^ and ^HA^Gγ_1_C71S^+/–^ ; Gγ_1_^−/−^ mice. An identical breeding strategy using Gα_t1_ knockout mice (Calvert et al., 2000) was used to generate ^HA^Gγ_1_^+/−^; Gα_t1_^−/−^ strain. All experiments involving mice were performed according to procedures approved by the Animal Care and Use Committee of West Virginia University.

### Dark Adaptation and Light Conditioning of Mice

For dark adaptation, mice were kept in their original cages in the dark room for at least 12 h, and from then on, all procedures were performed under dim red light. For light conditioning, animal’s pupils were dilated with a mixture of 1.25% phenylephrine hydrochloride and 0.5% tropicamide ophthalmic solution for 20 min, after which mice were exposed to diffused 5000 lux white light, while free running in a white box for 10 min. Subsequently, mice were euthanized and their eyes were harvested and fixed.

### Quantification of Proteins by Western Blotting

To quantify protein levels in isolated retinas, retina was dissected from the eye, gently cleaned from the contaminating tissues, and frozen on dry ice. Frozen retina was thawed and immediately homogenized by short ultrasonic pulses in 0.2 ml of urea sample buffer (USB) containing 125 mM Tris/HCl, pH 6.8, 4% SDS, and 6M urea. The extract was cleared by centrifugation. Total protein concentration was determined on a Nanodrop ND-1000 spectrophotometer, and all tested samples were adjusted to the lowest value. Prior to analysis by SDS PAGE, bromophenol blue tracking dye and 5% β-mercaptoethanol were added to each sample. To quantify Gα_t1_, a whole eye was enucleated and frozen on dry ice. Frozen eyes were homogenized in USB supplemented with bromophenol blue tracking dye and 5% β-mercaptoethanol by short ultrasonic pulses. The extracts were cleared by centrifugation. Equal aliquots of the compared samples were separated next to each other on a 10%–20% SDS PAGE, transferred to Immobilon FL membrane (Immunobilon-FL, Millipore, Billerica, MA, USA), and analyzed by Western blotting, using an Odyssey Infrared Imaging System (LI-COR Biosciences, Lincoln, NE, USA) according to the manufacturer’s protocol.

### Pull Down of Epitope-Tagged ^HA^Gγ_1_ and ^HA^Gγ_1_C71S

Retinas were dissected and homogenized in RIPA buffer (R0278, Sigma, St. Louis, MO, USA) by short ultrasonic pulses. Resulting retinal extracts were cleared by centrifugation, and the supernatant was incubated with washed Pierce anti-HA magnetic beads (88836, Thermo Fisher Scientific, Waltham, MA, USA) for 30 min at room temperature. Beads were washed four times for 3 min with RIPA buffer, after which the captured proteins were eluted with 3% ammonium hydroxide solution and vacuum-dried. For Western blot analysis, lyophilized samples were reconstituted in USB buffer. In phosducin co-precipitation assay, instead of RIPA buffer the pull downs were conducted in 10 mM Hepes/HCl, pH 7.0, 180 mM NaCl, 2% IGEPAL CA-630 (56741, Sigma, St. Louis, MO, USA) supplemented with Protease inhibitors cocktail set 1 (539131, Calbiochem).

### Triton X-114 Phase Partitioning

Method was modified from Justice et al. (1995). Retinal extract was prepared by homogenizing four retinas in 0.2 ml of Buffer A (PBS, protein inhibitor cocktail (Roche), bromophenol blue tracking dye), and clearing insoluble parts by centrifugation. 20 μl of 10% Triton X-114 (648468, Calbiochem) was added to 180 μl of the retinal extract, mixed by gentle inversion and pre-warmed to 37°C for 5 min. The sample was centrifuged (300× *g*, 10 min) at 37–40°C leading to a separation of aqueous and Triton X-114 layers, with the later becoming blue-colored due to migration of bromophenol blue into the detergent phase. The upper aqueous layer was collected in a new test tube and mixed with 20 μl of 10% Triton X-114. The lower Triton X-114 layer was mixed with 0.2 ml of Buffer A, to equalize the compositions and volumes of the two fractions. Then, 0.8 ml of RIPA buffer was added to each fraction, and the epitope-tagged Gγ_1_ was captured with anti-HA magnetic beads and analyzed by Western blotting, as described above.

### Immunofluorescence Microscopy

#### Frozen Retinal Cross-sections

Enucleated eye was immersed in freshly prepared 4% paraformaldehyde in PBS for 5 min at room temperature, and then the cornea was removed. The eye was fixed for additional 55 min, washed in PBS three times for 10 min, and incubated in 20% sucrose in PBS overnight at 4°C. The eye was then incubated in 1:1 mixture of 20% sucrose in PBS:OCT (Cryo Optimal Cutting Temperature Compound, Sakura) for 1 h. The lens was removed, and the resulting eyecup was positioned in a plastic tray with OCT, and flash frozen on a dry ice/ethanol bath. Sixteen micrometer thick cross-sections were cut on a Leica CM1850 Cryostat, and placed on Superfrost Plus slides (Fisher Scientific). Retinal sections mounted on slides were washed with PBS to remove OCT, and then blocked for 1 h in PBS containing 5% goat sera and 0.5% Triton X-100. Primary and secondary antibodies were diluted in PBS containing 2.5% goat sera and 0.5% Triton X-100. Incubation with primary antibodies typically lasted overnight at room temperature. Slides were washed two times for 15 min with PBS containing 0.1% Triton X-100, prior to the incubation with secondary antibodies, Alexa Fluor 568 goat anti-rat (A11077, Life Technologies) and Alexa Fluor 488 donkey anti-rabbit (A21206, Invitrogen), and 4′,6-diamindino-2-phenylindole dihydrocholide (DAPI; Roche, Indianapolis, IN, USA) nuclear stain, for 1 h. Slides were washed three times for 10 min with PBS containing 0.1% Triton X-100, mounted with ProLong Gold (Life Technologies), and cover slipped. Images were acquired on a Zeiss LSM 510 and Nikon C2 confocal microscopes, and processed using a NIC Elements Imaging Software.

#### Flat-Mounted Retina

The whole eye was fixed in 4% paraformaldehyde in PBS for 5 min at room temperature, the anterior segment was removed, and the retina was extracted. The retina was cleaned from contaminating tissues and fixed in the same solution for 6 h. The fixed retina was washed three times for 30 min with PBS, blocked for 4 h in PBS containing 5% goat sera and 0.5% Triton X-100, and probed with primary and secondary antibodies, which were prepared as described above, for at least 14 h. The washes after applying each antibody were three times for 30 min with PBS containing 0.1% Triton X-100. The washed retina was positioned on Superfrost Plus slides (Fisher Scientific) with the outer segments of the photoreceptors facing up. Four equally placed radial cuts were made to flatten the retina, and then the retina was mounted, and cover slipped for imaging as described above.

### Electroretinography (ERG)

Mice were dark-adapted overnight prior to testing, and all procedures were performed under dim red light. During recording, mice were anesthetized by breathing 1.5% isoflurane with 2.5 liters per minute (lpm) oxygen through a nose cone, while lying on a heated platform. The animal’s pupils were dilated with a mixture of 1.25% phenylephrine hydrochloride and 0.5% tropicamide ophthalmic solution. A reference needle electrode was inserted under the loose skin between the ears. Flash Electroretinography (ERG) responses were recorded from both eyes with custom-made silver wire electrodes positioned on each cornea, with contact being made with a drop of hypromellose solution (2% hypromellose in PBS; Gonioscopic Prism Solution, Wilson Ophthalmic, Mustang, OK, USA). The recordings were performed on the UTAS Visual Diagnostic System with BigShot Ganzfeld, UBA-4200 amplifier and interface, and EMWIN 9.0.0 software (LKC Technologies, Gaithersburg, MD, USA).

### Experimental Design and Statistical Analyses

Each confocal microscopy experiment was repeated at least three times to ensure that the results were reproducible from animal to animal and also between the slides. The representative images with the most commonly observed staining patterns and unperturbed morphology, usually originating from central retina in immediate vicinity to the optic nerve, were selected for the figures. In all quantifications (except for Figure 2A), the significance level was determined using the independent two-tailed Student’s* t*-test, and the values were expressed as mean ± SEM. The light-sensitivity curves of ERG a-wave (Figure 2A) were compared using Friedman repeated measures analysis of variance (ANOVA) on ranks.

**Figure 1:**
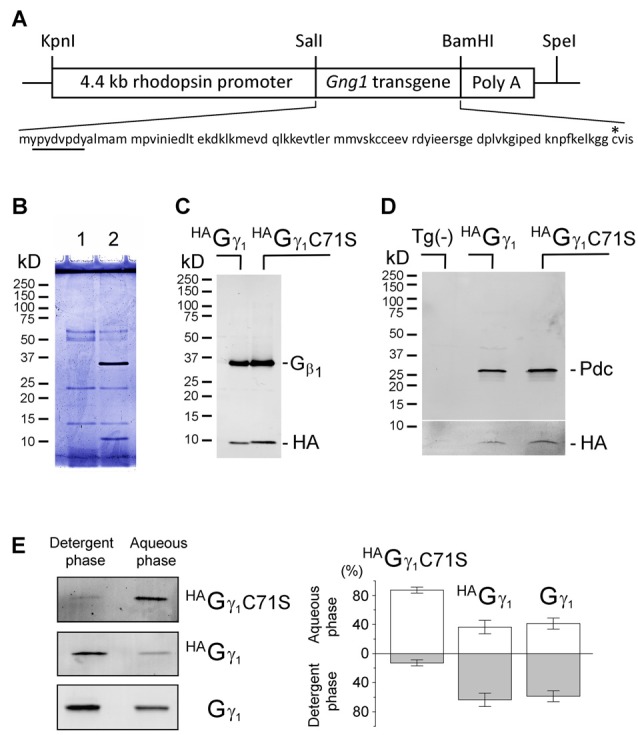
Assembly of transgenic ^HA^Gγ_1_ and ^HA^Gγ_1_C71S with the endogenous Gβ_1_ in retinal photoreceptors. **(A)** Transgenic construct used for generation of ^HA^Gγ_1_ mice and amino acid sequence of the encoded protein with HA-tag (underlined) and C71 (asterisk). **(B)** SDS polyacrylamide gel stained with Coomassie blue showing anti-HA pull downs from 10 retinas of the transgene-negative (1) and transgene-positive (2) ^HA^Gγ_1_ mice. **(B)** Western blot analysis of anti-HA pull downs from the retinas of ^HA^Gγ_1_ and ^HA^Gγ_1_C71S mice with antibodies against Gβ_1_ and HA. **(C)** Representative Western blot showing co-precipitation of phosducin (Pdc) with ^HA^Gγ_1_ or ^HA^Gγ_1_C71S (HA). Tg(-) indicates transgene-negative littermates. The break separates two blots that were adjusted differently. Identical amounts of phosducin co-precipitated with ^HA^Gγ_1_ and ^HA^Gγ_1_ C71S (*n* = 4). **(E)** Representative experiments illustrating partitioning of ^HA^Gγ_1_C71S, ^HA^Gγ_1_, and endogenous Gγ_1_ between the detergent (Triton X-114) and aqueous phases. Specific bands were visualized by Western blotting with anti-HA (^HA^Gγ_1_C71S, ^HA^Gγ_1_) and anti-Gγ_1_ (Gγ_1_). **(D)** Average distribution of each protein in the aqueous and detergent phases (^HA^Gγ_1_C71S: 87/13 ± 4%; ^HA^Gγ_1_: 36/64 ± 9%, Gγ_1_: 41/59 ± 8%, error bars are SEM, *n* = 4).

**Figure 2:**
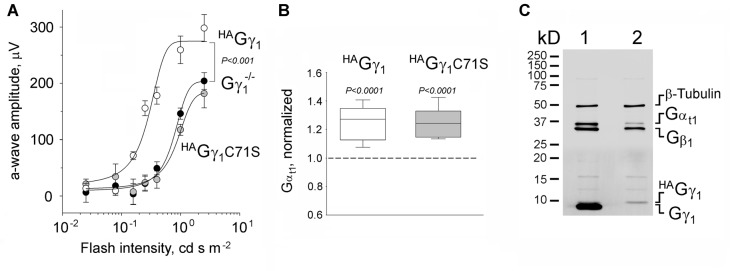
Functional rescue of Gγ_1_-null rod photoreceptors by ^HA^Gγ_1_ and ^HA^Gγ_1_C71S. **(A)** Visual responses of rods were analyzed by electroretinography (ERG) and the amplitude of maximum elicited a-wave was plotted as a function of flash intensity. One month old ^HA^Gγ_1_ mice (white circles), ^HA^Gγ_1_C71S mice (gray circles) mice of Gγ_1_^−/−^ background were compared with their transgene-negative Gγ_1_^−/−^ littermates (black circles) as negative control. Error bars are SEM, *n* = 6. Each data set was fitted with a sigmoidal three parameter function, and the resulting light-sensitivity curves were analyzed by repeated measures analysis of variance (ANOVA). **(B)** Median levels of Gα_t1_ in rod photoreceptors of ^HA^Gγ_1_ (white box) and ^HA^Gγ_1_C71S (gray box) mice of Gγ_1_^−/−^ background at the age of 1 month. Gα_t1_ was quantified in whole eye extracts by Western blotting, and the values in transgene-positive littermates were normalized to those in transgene-negative littermates, analyzed on the same blot. On average, the level of Gα_t1_ was increased by 25 ± 3% in ^HA^Gγ_1_ mice and by 24 ± 3% in ^HA^Gγ_1_C71S mice, compared to their transgene-negative siblings (SEM, *n* = 11, two-tailed *P*-value). **(C)** Representative Western blot showing the amounts of transducin Gα_t1_ Gβ_1_ and Gγ_1_ subunits, and β-Tubulin, in whole retinal extracts of ^HA^Gγ_1_^+/−^; Gγ_1_^+/−^ (1) and ^HA^Gγ_1_^+/−^; Gγ_1_^−/−^ (2) littermates.

### Antibodies

Proteins were detected using the following antibodies: rat anti-HA antibody (11867423001, Roche, Indianapolis, IN, USA), rabbit anti-HA antibody (sc-805, Santa Cruz Biotechnology, Dallas, TX, USA), rabbit anti-Gα_t1_ (sc-389, Santa Cruz Biotechnology), rabbit anti-Gβ (sc-378, Santa Cruz Biotechnology), rabbit anti-Gγ_1_ (sc-373, Santa Cruz Biotechnology), mouse anti-β-tubulin (T0198, Sigma), rabbit anti-syntaxin 3 (15556, Proteintech Group), rabbit anti-peripherin/rds (a gift from Dr. Kathleen Boesze-Battaglia, University of Pennsylvania, Philadelphia, PA, USA), mouse anti-arrestin (C10C10) (a gift from Dr. Wesley Clay Smith, University of Florida, Gainesville, FL, USA).

## Results

### Integration of ^HA^Gγ_1_ and ^HA^Gγ_1_C71C into Transducin Pool of Rod Photoreceptors

The HA-tagged γ subunit of transducin (^HA^Gγ_1_) and its mutant carrying a cysteine-to-serine substitution at position 71 (^HA^Gγ_1_C71S) were expressed from transgenes controlled by the retinal rod photoreceptor-specific rhodopsin promoter (Figure 1A). Overexpressing ^HA^Gγ_1_ and ^HA^Gγ_1_C71S in mouse rods did not have an obvious negative effect on these cells. Each of these epitope-tagged proteins could be captured from retinas homogenized in RIPA buffer with anti-HA magnetic beads. These anti-HA pull downs always contained the ~10 kD ^HA^Gγ_1_ band and a 35 kD band (Figure 1B). The 35 kD band was identified as Gβ_1_ by Western blotting (Figure 1C). When this assay was conducted in a milder non-ionic detergent, the pull downs also contained phosducin (Figure 1D), an abundant rod phosphoprotein that forms a complex with Gβγ dimers (Gaudet et al., 1996). Thus, each transgenic protein formed a dimer with endogenous Gβ_1_ and the resulting Gβγ dimers were capable of binding to phosducin *in vivo*. The hydrophobicity of these chimeric Gβγ dimers was assessed based on their partitioning between the aqueous and detergent Triton X-114 phases (Figure 1E; Supplementary Figure S1). We found that Gβ_1_^HA^Gγ_1_C71S predominantly partitioned to the aqueous phase, while a larger fraction of the Gβ_1_^HA^Gγ_1_ was retained by the detergent phase. Gβ_1_^HA^Gγ_1_ partitioning was similar to the partitioning of endogenous Gβ_1_γ_1_. We attributed the diminished hydrophobicity of Gβ_1_^HA^Gγ_1_C71S to the deletion of the farnesylation site.

### Rescue of Gγ_1_ Knockout Mice by ^HA^Gγ_1_ and ^HA^Gγ_1_C71S

To evaluate the functional efficiency of ^HA^Gγ_1_ and ^HA^Gγ_1_C71S, the corresponding transgenic lines were backcrossed into a Gγ_1_ knockout background. Gγ_1_ knockout mice have been shown to display diminished rod responses measured by ERG (Kolesnikov et al., 2011). Therefore, we comparatively analyzed ^HA^Gγ_1_ and ^HA^Gγ_1_C71S mice of Gγ_1_^−/−^ background vs. their transgene-negative littermates by ERG. We focused on the amplitude of the a-wave, since it is directly generated by mass rod response (Figure 2A; Supplementary Figure S2). Our analysis revealed that Gγ_1_^−/−^ mice expressing ^HA^Gγ_1_ produced significantly larger ERG a-waves than their transgene-negative siblings across a wide range of stimuli (Figure 2A, compare white and black circles). This rescue of the a-wave appeared to be partial, because its maximum amplitude of 250 μV was about half of an average WT value (Supplementary Figure S2). No ERG a-wave increase was observed in ^HA^Gγ_1_C71S-expressing mice (Figure 2A, compare gray and black circles).

Rod photoreceptors of Gγ_1_ knockout mice fail to elicit normal ERG responses due to a major destabilization of transducin, a heterotrimeric G protein that mediates visual signaling. As such, the level of transducin Gα_t1_ subunit in Gγ_1_-null rods declines by more than 5-fold (Lobanova et al., 2008; Kolesnikov et al., 2011), and was 6.1 ± 0.9% (*n* = 3, SEM) of the WT level in the used Gγ_1_^−/−^ strain (data not shown). To test the ability of ^HA^Gγ_1_ and ^HA^Gγ_1_C71S to rescue this phenotype, we compared the level of Gα_t1_ in ^HA^Gγ_1_ and ^HA^Gγ_1_C71S mice of Gγ_1_^−/−^ background to that of their transgene-negative littermates by Western blotting. Given that Gα_t1_ is exclusively expressed in rod photoreceptors, the analysis was conducted in whole eye extracts. We found that both transgenes, ^HA^Gγ_1_ and ^HA^Gγ_1_C71S increased the level of Gα_t1_ in the retina of Gγ_1_ knockout mice by ~25% of the base level of Gα_t1_ in our Gγ_1_^−/−^ strain (Figure 2B), which corresponds to 1.5% increase compared to the WT level of Gα_t1_.

While breeding these mice, we estimated how much of ^HA^Gγ_1_ is expressed from the transgene compared to the endogenous Gγ_1_. For that, whole retinal extracts were prepared from ^HA^Gγ_1_ littermates of Gγ_1_^−/−^ and Gγ_1_^+/−^ backgrounds. Aliquots of these extracts, containing equal amounts of total protein, were analyzed by Western blotting using antibody against all three transducin subunits with β-Tubulin, as a loading control (Figure 2C). The amount of transgenic ^HA^Gγ_1_ in the retina was much lower than the endogenous Gγ_1_ level, which made a direct comparison of both bands inaccurate due to the non-linearity of Western blotting. To overcome this limitation, we compared retinal extracts of ^HA^Gγ_1_^+/−^; Gγ_1_^−/−^ mice with WT retinal extracts, which was diluted with Gγ_1_-null extracts. Our goal was to find a dilution at which both signals would match. Each specific band was detected with anti-Gγ_1_, which recognizes the same epitope within ^HA^Gγ_1_ and Gγ_1_. We found that when a WT retinal extract was diluted by 100-fold, both signals began to match, which thus provided an accurate estimation that transgenic ^HA^Gγ_1_ was expressed at about 1.2 ± 0.3% (*n* = 3, SEM) of the endogenous Gγ_1_. The level of ^HA^Gγ_1_ remained the same in ^HA^Gγ_1_ mice of WT background.

**Figure 3:**
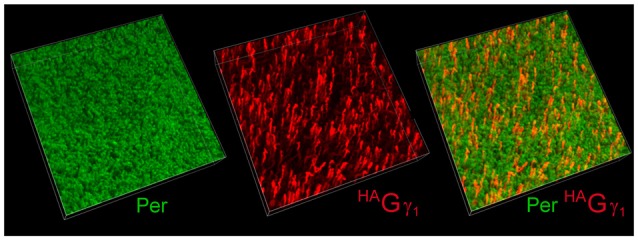
Mosaic expression of the transgene in the retina. Flat-mounted retina from ^HA^Gγ_1_ mouse was analyzed by immunofluorescence confocal microscopy. Rod outer segments (OSs) were visualized with antibody against peripherin/rds (Per, green), and epitope-tagged ^HA^Gγ_1_ was visualized with antibody against HA (red). In the shown 100 μm × 100 μm × 20 μm z-stack, about 9% of rods are estimated to express ^HA^Gγ_1_.

The low abundance of ^HA^Gγ_1_ in the retina was generally consistent with the mosaic expression of the transgene. To determine the percentage of rods that expressed our transgene, we visualized ^HA^Gγ_1_-expressing rods in flat-mounted retinal preparation with antibody against HA by immunofluorescent confocal microscopy (Figure 3, red). Their number was compared with the total number of rod cells visualized with peripherin/rds (Figure 3, green). According to our estimation the transgene was expressed in 9% of rods. Thus, the level of ^HA^Gγ_1_ in each transgene-positive rod was about 10% of the endogenous Gγ_1_ level.

### Subcellular Localization of ^HA^Gγ_1_ and ^HA^Gγ_1_C71S under Different Conditions of Illumination

**Figure 4:**
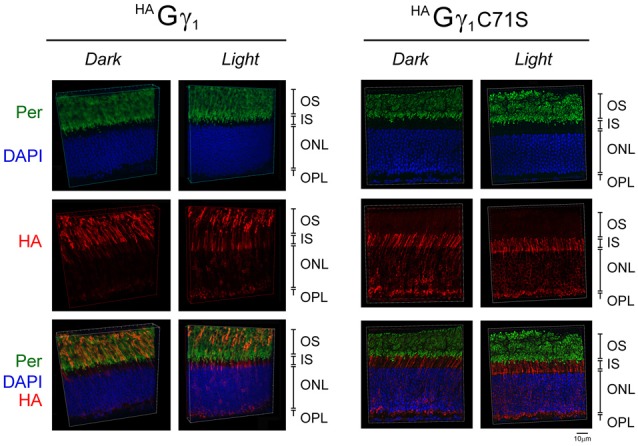
Subcellular localization of ^HA^Gγ_1_ and ^HA^Gγ_1_ C71S under different conditions of illumination. ^HA^Gγ_1_ and ^HA^Gγ_1_C71S mice were either dark-adapted overnight (*Dark*) or light-conditioned for 10 min (*Light*). Protein localization was determined in frozen retinal cross-sections by immunofluorescence confocal microscopy. Peripherin/rds (Per, green) was utilized as the OS marker, ^HA^Gγ_1_ and ^HA^Gγ_1_C71S were visualized with antibody against HA-tag (red), and photoreceptors nuclei (blue) were stained with DAPI. The indicated retinal layers are: OS, outer segments layer; IS, inner segments layer; ONL, outer nuclear layer containing rod nuclei; OPL, outer plexiform layer containing rod synapses.

Vertebrate rods display a unique physiological response to saturating levels of light including the translocation of transducin from the rod outer segment to other compartments of these cells (Artemyev, 2008). To test whether ^HA^Gγ_1_ re-distributes along with transducin, we examined its subcellular localization under different conditions of illumination (Figure 4). The epitope-tagged ^HA^Gγ_1_ was readily detectable in retinal cross-sections by immunofluorescence confocal microscopy with the antibody against HA tag. We confirmed that this protein was only expressed in a fraction of rods, however this mosaicism often provided an unobstructed view of individual rod cells. This view would have been impossible otherwise due to a high density and significant overlap of these neurons in the retina.

Our analyses revealed that in dark-adapted mice ^HA^Gγ_1_ appeared predominantly in rod OSs, where it co-localized with the outer segment marker peripherin/rds (Figure 4). In mice exposed to bright ambient light for 10 min, ^HA^Gγ_1_ immunoreactivity declined in rod outer segments and increased in other cellular compartments, including the inner segment, cytoplasm around the nucleus, and the synapse (Figure 4). In agreement with previous report (McGinnis et al., 2002), the base of the outer segment adjacent to the inner segment appeared to lose the ^HA^Gγ_1_ signal first. Such subcellular distribution of ^HA^Gγ_1_ supported a notion that its chimeric Gβγ dimers accumulated in the rod outer segment in the dark, and moved away from this compartment during sustained light exposure. This data further strengthened our conclusion that epitope-tagged ^HA^Gγ_1_ expressed from a transgene became fully integrated into rod photoreceptors’ transducin pool.

**Figure 5:**
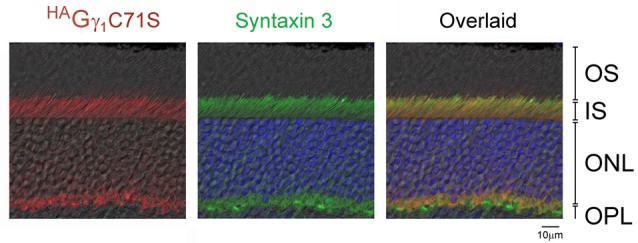
Exclusion of ^HA^Gγ_1_C71S and syntaxin 3 from rod OSs. Retina of dark-adapted ^HA^Gγ_1_C71S mice were analyzed by immunofluorescence confocal microscopy. The immunostaining of ^HA^Gγ_1_C71S (red) and syntaxin 3 (green) are shown together with DIC image. Photoreceptors nuclei are stained with DAPI. The indicated retinal layers are: OS, outer segments layer; IS, inner segments layer; ONL, outer nuclear layer containing rod nuclei; OPL, outer plexiform layer containing rod synapses.

**Figure 6:**
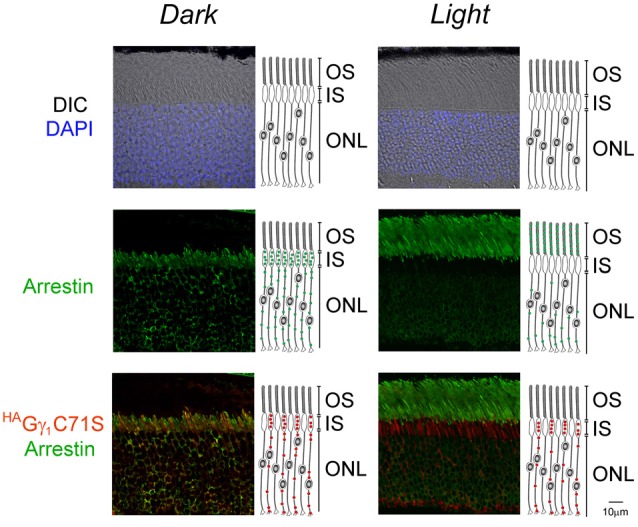
Normal arrestin translocation in rods of ^HA^Gγ_1_C71S mice. ^HA^Gγ_1_C71S mice were either dark-adapted overnight (*Dark*) or light-conditioned for 10 min (*Light*) and their retinas were analyzed by immunofluorescence confocal microscopy. The immunostaining of ^HA^Gγ_1_C71S (red), arrestin (green), and the DIC image with photoreceptors nuclei stained with DAPI (blue) are shown. Cartoon illustrates localization of ^HA^Gγ_1_C71S (red circles) and arrestin (green circles) in rod cells. The indicated retinal layers are: OS, outer segments layer; IS, inner segments layer; ONL, outer nuclear layer containing rod nuclei.

Similar to ^HA^Gγ_1_, transgenic ^HA^Gγ_1_C71S was clearly visible in rods. In contrast to ^HA^Gγ_1_, this protein was found to be excluded from rod outer segment under all conditions of illumination. For example, even after 12 h of dark adaptation rod outer segments contained virtually no ^HA^Gγ_1_C71S (Figure 4). The distribution of ^HA^Gγ_1_C71S in rods was identical to that of syntaxin 3 (Protein: syntaxin 3, gene: *Stx3/Syn-3*; Figure 5), whose segregation from the outer segments has been attributed to the gating function of photoreceptor’s cilium (Datta et al., 2015). For that reason, we first hypothesized that Gβγ dimers comprised of non-farnesylated ^HA^Gγ_1_C71S may be excluded from the rod outer segment due to the gating function of the cilium. However, we also monitored subcellular localization of arrestin (protein: visual arrestin 1, gene: *Arr1/Sag*), a phototransduction protein that undergoes translocation in the opposite direction of transducin, i.e., arrestin withdraws from the cell body and accumulates in the rod outer segment upon light stimulation (Broekhuyse et al., 1985; Philp et al., 1987; Mangini and Pepperberg, 1988; Whelan and McGinnis, 1988; also reviewed in Pearring et al., 2013). We observed unobstructed translocation of arrestin in all examined rods, indicating that this non-lipidated soluble protein comparable to a Gβγ dimer in size moves freely through connecting cilium, while ^HA^Gγ_1_C71S remains blocked (Figure 6). This result is generally incompatible with a notion the photoreceptor’s cilium indiscriminately obstructs the diffusion of all soluble proteins, including non-farnesylated Gβγ.

### Transducin α Subunit Is Required for the Accumulation of ^HA^Gγ_1_ in Rod Outer Segments

**Figure 7:**
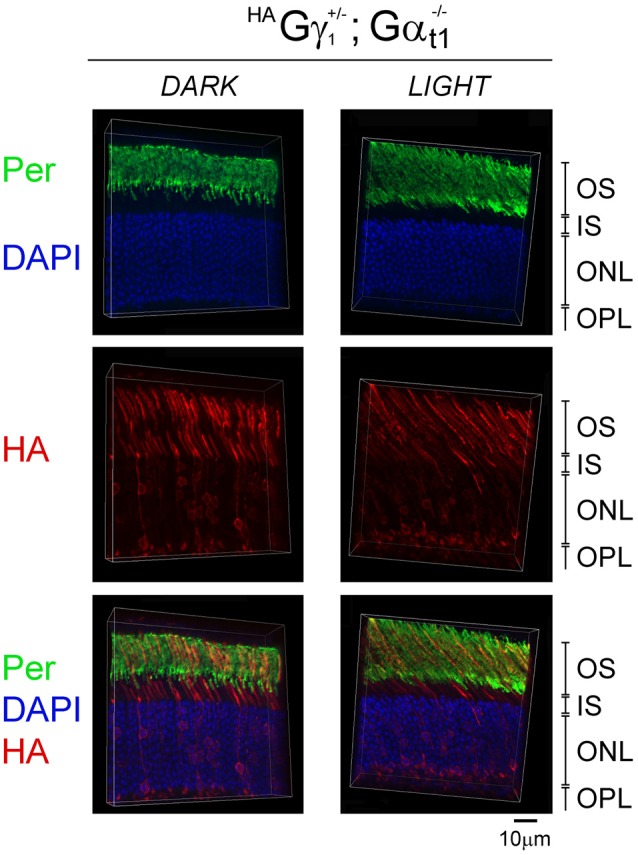
Subcellular localization of ^HA^Gγ_1_ in the absence of transducin α subunit. ^HA^Gγ_1_ mice of Gα_t1_^−/–^ background were either dark-adapted overnight (*DARK*) or light-conditioned for 10 min (*LIGHT*). Protein localization was determined in frozen retinal cross-sections by immunofluorescence confocal microscopy. Peripherin/rds (Per, green) was utilized as the OS marker, ^HA^Gγ_1_ (red) was visualized with antibody against HA-tag, and photoreceptors nuclei (blue) were stained with DAPI. The indicated retinal layers are: OS, outer segments layer; IS, inner segments layer; ONL, outer nuclear layer containing rod nuclei; OPL, outer plexiform layer containing rod synapses.

To determine the role of the transducin α subunit (protein: Gα_t1_, gene: *Gnat*) in the trafficking of ^HA^Gγ_1_ to the rod OS, ^HA^Gγ_1_ mice were backcrossed into Gα_t1_-null background, which eliminated the expression of transducin α subunit (Calvert et al., 2000). In the absence of Gα_t1_, endogenous Gβ_1_γ_1_ spreads throughout the rod photoreceptor and does not undergo light-driven translocation (Zhang et al., 2003; Lobanova et al., 2008; Belcastro et al., 2012). When we analyzed subcellular localization of ^HA^Gγ_1_ in the dark-adapted Gα_t1_-null rods, we found this protein in all compartments of rod cells, including the inner segment, cytoplasm around the nuclei, and the synapse. At the same time, a significant amount of ^HA^Gγ_1_ remained in the rod outer segment regardless of the different conditions of illumination (Figure 7). These data further demonstrate that Gβγ dimers comprised of transgenic ^HA^Gγ_1_ fully emulate the properties of endogenous Gβ_1_γ_1_ and that they could serve as a surrogate for endogenous Gβ_1_γ_1_.

### ^HA^Gγ_1_ Enhances Targeting of the Transducin α Subunit in the Rod Outer Segment

**Figure 8:**
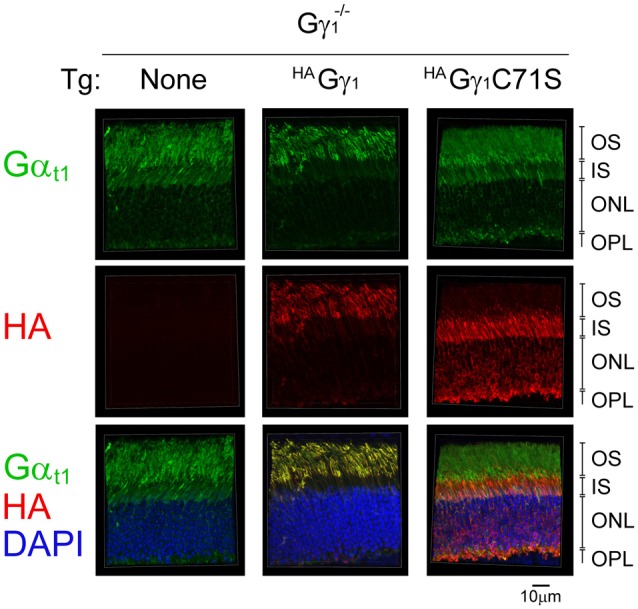
The effect of ^HA^Gγ_1_ and ^HA^Gγ_1_C71S on the localization of transducin α subunit to the rod OS in the dark. Mice of Gγ_1_^−/−^ background expressing indicating transgene (Tg) were dark-adapted overnight and protein localization was determined in frozen retinal cross-sections by immunofluorescence confocal microscopy. Transducin α subunit (green) was detected with antibodies against Gα_t1_, epitope-tagged ^HA^Gγ_1_ and ^HA^Gγ_1_C71S (red) were visualized with antibody against HA, and photoreceptors nuclei (blue) were stained with DAPI. The indicated retinal layers are: OS, outer segments layer; IS, inner segments layer; ONL, outer nuclear layer containing rod nuclei; OPL, outer plexiform layer containing rod synapses.

Back-crossing ^HA^Gγ_1_ and ^HA^Gγ_1_C71S mice into a Gγ_1_-null background allowed us to evaluate whether farnesylated and non-farnesylated Gγ could target endogenous transducin Gα to the rod OS. For each strain, we compared subcellular localization of Gα_t1_ in the dark-adapted transgene-negative and transgene-positive mice. In rods of transgene-negative Gγ_1_-null mice that do not express any Gγ, and therefore cannot assemble Gβγ dimer (Kolesnikov et al., 2011), Gα_t1_ was present in all rod cellular compartments (Figure 8). This result is consistent with previous work that found a similar subcellular distribution of Gα_t1_ in rods of Gγ_t1_-null mice, which was determined by Western blot analysis of serial tangential sections of the retina (Lobanova et al., 2008). In contrast to Gγ_1_-null mice, Gα_t1_ in ^HA^Gγ_1_-expressing rods was found predominantly in rod outer segments where it co-localized with ^HA^Gγ_1_ (Figure 8). However, in ^HA^Gγ_1_C71S-expressing rods the localization of Gα_t1_ remained the same as in the transgene-negative mice (Figure 8). These results demonstrate that farnesylated ^HA^Gγ_1_ enhances targeting of Gα_t1_ to the outer segment of Gγ_1_-null rods, the same as WT Gγ_1_(Lobanova et al., 2008), whereas non-farnesylated ^HA^Gγ_1_C71S appeared to lack this ability. They also support a notion that Gα_t1_ is unable to shuttle non-farnesylated Gβγ dimers into the cilium, which is evident from a complete exclusion of ^HA^Gγ_1_C71S from rod outer segments (Figure 8).

## Discussion

### Farnesylation Controls Ciliary Localization of Gβγ Dimer

We found that epitope-tagged ^HA^Gγ_1_ and ^HA^Gγ_1_C71S readily dimerizes with endogenous transducin Gβ_1_ subunit *in vivo*, which further supports the idea that prenylation of a Gγ is not required for its dimerization with Gβ (Simonds et al., 1991; Pronin and Gautam, 1993; Higgins and Casey, 1994). Interestingly, ^HA^Gγ_1_ and ^HA^Gγ_1_C71S were equally competent to rescue the expression of Gα_t1_ in rod photoreceptors of Gγ_1_ knockout mice (Figure 2B). Given that Gα_t1_ becomes destabilized in this mouse model due to a posttranslational mechanism (Lobanova et al., 2008; Kolesnikov et al., 2011), our result suggests that either Gβ_1_^HA^Gγ_1_ or Gβ_1_^HA^Gγ_1_C71S could form a transducin heterotrimer. This result is consistent with the ability of the purified Gβ_1_γ_1_ that was subjected to endo-Lys-C proteolysis, which removes the farnesyl modification, to form a heterotrimeric G protein *in vitro* (Lambright et al., 1996). However, only ^HA^Gγ_1_ restored rod visual responses of Gγ_1_-null mice, whereas ^HA^Gγ_1_C71S did not have a physiological effect (Figure 2A). The functional deficiency of ^HA^Gγ_1_C71S was explained by its exclusion from rod outer segments (Figures 5, 6), indicating that any G protein comprised of ^HA^Gγ_1_C71S remained spatially separated from its coupled receptor, rhodopsin. The exclusion of ^HA^Gγ_1_C71S was likely caused by the inability of ^HA^Gγ_1_C71S to undergo farnesylation. This conclusion is supported by the observation that the N-acylation-deficient Gα_t1_ predominantly localizes in the inner compartments of rod instead of the outer segment (Kerov et al., 2007). Furthermore, eliminating endoproteolysis and c-terminal methylation by a knockout of RAS-converting enzyme 1 in rods has no effect on the accumulation of Gγ_1_ in rod OSs, although farnesylation is a prerequisite for both steps (Christiansen et al., 2011). However, the last three amino acids (VIS) that are removed by endoproteolysis in ^HA^Gγ_1_ and endogenous Gγ_1_ are present on ^HA^Gγ_1_C71S. Nevertheless, we attribute the mislocalization of ^HA^Gγ_1_C71S and the lack of rescue in Gγ_1_-null mice to the lack of farnesylation, since the farnesyl group contributes more to the hydrophobicity of Gγ_1_ than the last three amino acids.

### Rod Outer Segment Is Accessible for the Individual Gα and Gβγ Subunits

We have observed the reciprocal requirement of Gα_t1_ and Gβ_1_γ_1_ subunits for their efficient ciliary sequestration in resting rods. Using two knockout mouse models in which Gα_t1_ or Gβ_1_γ_1_ was ablated, we have determined subcellular localization of each individual subunit when its partner was absent. Epitope-tagged Gβ_1_^HA^Gγ_1_, which was used as a surrogate for Gβ_1_γ_1_, was clearly visible in rod outer segments of Gα_t1_ knockout mice (Figure 7). Gα_t1_ was present in outer segments of Gγ_1_-null rods lacking Gβ_1_γ_1_ dimer (Figure 8). Our results further confirm the conclusion of previous studies that the accumulation of transducin in outer segments of dark-adapted rods is based on G protein heterotrimer assembly (Zhang et al., 2003; Lobanova et al., 2008). Yet, it is important to note that together these results disagree with the notion that assembly of heterotrimeric G protein is a prerequisite for ciliary trafficking of transducin in rods (Zhang et al., 2003). Instead these results suggest that the Gα_t1_ subunit and Gβ_1_γ_1_ dimer could each access the rod outer segment as an individual entity.

### What Drives Ciliary Exclusion of Non-farnesylated Gβγ Dimer?

Our most surprising observation from these studies was the strong exclusion of non-farnesylated Gβ_1_^HA^Gγ_1_C71S from rod OSs, where this protein remained below the level of detectability for immunofluorescence confocal microscopy under all tested conditions (Figures 4, 5). This conclusion was further supported by non-farnesylated Gβ_1_^HA^Gγ_1_C71S’s failure to rescue light responses elicited by the outer segments of Gγ_1_-null rods (Figure 2A). However, non-farnesylated Gβ_1_^HA^Gγ_1_C71S was capable of increasing the levels of Gα_t1_ likely by forming heterotrimeric transducin elsewhere in the cells (Figure 2B). Below, we describe two mechanisms that could explain why non-farnesylated Gβ_1_^HA^Gγ_1_C71S was excluded from rod OSs.

Some evidence demonstrates that subcellular translocation of transducin is aided by two “trafficking chaperones” that bind transducin α and βγ subunits and conceal their lipid groups. Those include Unc119 that acts a sheath for the N-terminal myristoyl group of Gα_t1_ (Gopalakrishna et al., 2011), and phosducin that binds free Gβ_1_γ_1_. When phosducin forms a complex with Gβ_1_γ_1_, the farnesyl moiety of Gγ_1_ becomes buried in between the β-propeller blades of Gβ_1_ (Loew et al., 1998). Deletion of Unc119 or phosducin was shown to affect the accumulation of transducin in rod outer segments (Sokolov et al., 2004; Zhang et al., 2011), which suggests that transducin’s subunits are likely to diffuse in this compartment as soluble Unc119/Gα_t1_ and Gβ_1_γ_1_/phosducin complexes. In light of these data, the inability of soluble non-farnesylated Gβ_1_^HA^Gγ_1_C71S to enter the outer segment seems rather counterintuitive. In fact, this result is more consistent with a different mechanism proposed by Baehr and colleagues (Karan et al., 2008) that is based on intraflagellar transport (IFT). According to this mechanism, transducin passes through the connecting cilium and enters the outer segment as a membrane cargo pulled along axonemal microtubules by motor proteins. The IFT mechanism would explain a prerequisite for the Gβγ lipid modification—without a farnesyl group Gβγ would not be able to anchor itself to the ciliary membrane. It is also consistent with the observation that swapping the farnesyl group of Gγ_1_ to a more hydrophobic geranylgeranyl does not alter the targeting of Gβ_1_γ_1_ to the rod outer segment (Kassai et al., 2005). Yet, it does not explain why non-farnesylated Gβγ remains excluded from the OS. Two mechanisms of exclusion are plausible.

The first mechanism is based on the premise that the connecting cilium comprises of a gate that hinders diffusion of soluble proteins. This is exemplified by the alleged gatekeeping action of centrins, which have been proposed to form dynamic oligomers with Gβ_1_γ_1_, regulated by Ca^2+^ and phosphorylation by casein kinase 2 in the connecting cilium of rod photoreceptors (Giessl et al., 2006; Trojan et al., 2008). This mechanism predicts that soluble proteins cannot pass through the gate if closed, however visual arrestin, a soluble protein lacking any lipid modification, moves to rod OSs, while Gβ_1_^HA^Gγ_1_C71S remains blocked (Figure 6). Therefore, we favor an alternative mechanism, in which soluble Gβ_1_^HA^Gγ_1_C71S does access the OS, and then becomes expelled from this compartment due to the steric volume exclusion effect demonstrated by Calvert and colleagues (Najafi and Calvert, 2012; Najafi et al., 2012).

According to it, the presence of a soluble protein in the rod outer segment inversely depends on its size due to highly constrained cytoplasmic space between outer segment disc membranes. Furthermore, the authors specifically proposed that transducin Gβ_1_γ_1_ could be expelled from rod outer segments upon binding to phosducin, because the Gβ_1_γ_1_/phosducin complex is two times larger than Gβ_1_γ_1_ alone (Najafi et al., 2012). It is plausible that non-farnesylated Gβ_1_^HA^Gγ_1_C71S cannot efficiently dissociate from a complex with phosducin, and therefore remains excluded from the OS. Some evidence support this hypothesis. For example, it was demonstrated that eliminating S54 and S71 phosphorylation sites of phosducin that promote dissociation of the Gβ_1_γ_1_/phosducin complex significantly hindered trafficking of transducin to the rod outer segments (Belcastro et al., 2012). Empirical testing of this role of phosducin remains the goal of subsequent investigation.

## Author Contributions

CB and MS: designed the studies, generated mouse models, conducted experiments, analyzed data and wrote the article. MB: generated mouse models. JM, DH and SK: conducted experiments. OK: generated mouse model and wrote the article.

## Conflict of Interest Statement

The authors declare that the research was conducted in the absence of any commercial or financial relationships that could be construed as a potential conflict of interest.

## Supplementary Material

FIGURE S1 Full blots from in Figure 1E illustrating partitioning of ^HA^Gγ_1_C71S, ^HA^Gγ_1_, and endogenous Gγ_1_ between the detergent (Triton X-114) and aqueous phases. Specific bands were visualized by Western blotting with anti-HA (^HA^Gγ_1_C71S, ^HA^Gγ_1_) and anti-Gγ_1_ (Gγ_1_).

FIGURE S2 Representative electroretinography (ERG) recordings from Figure 2A. Visual responses were obtained using Gγ_1_^−/−^(^HA^Gγ_1_^−/−^; Gγ_1_^−/−^), ^HA^Gγ_1_ (^HA^Gγ_1_^+/−^; Gγ_1_^−/−^), and ^HA^Gγ_1_C71S (^HA^Gγ_1_C71S^+/–^; Gγ_1_^−/−^) mice stimulated by flashes of indicated strength. Red trace is a typical response of a wild type (WT) mouse to a saturating 2.5 cd s m^−2^ flash.

## References

[B1] Artemyev N. O. (2008). Light-dependent compartmentalization of transducin in rod photoreceptors. Mol. Neurobiol. 37, 44–51. 10.1007/s12035-008-8015-2 18425604

[B2] Belcastro M. Song H. Sinha S. Song C. Mathers P. H. Sokolov M. (2012). Phosphorylation of phosducin accelerates rod recovery from transducin translocation. Invest. Ophthalmol. Vis. Sci. 53, 3084–3091. 10.1167/iovs.11-8798 22491418 PMC3382380

[B3] Brann M. R. Cohen L. V. (1987). Diurnal expression of transducin mRNA and translocation of transducin in rods of rat retina. Science 235, 585–587. 10.1126/science.3101175 3101175

[B4] Broekhuyse R. M. Tolhuizen E. F. Janssen A. P. Winkens H. J. (1985). Light induced shift and binding of S-antigen in retinal rods. Curr. Eye Res. 4, 613–618. 10.3109/02713688508999993 2410196

[B5] Burgoyne T. Meschede I. P. Burden J. J. Bailly M. Seabra M. C. Futter C. E. (2015). Rod disc renewal occurs by evagination of the ciliary plasma membrane that makes cadherin-based contacts with the inner segment. Proc. Natl. Acad. Sci. U S A 112, 15922–15927. 10.1073/pnas.1509285113 26668363 PMC4702997

[B6] Calvert P. D. Krasnoperova N. V. Lyubarsky A. L. Isayama T. Nicoló M. Kosaras B.. (2000). Phototransduction in transgenic mice after targeted deletion of the rod transducin α -subunit. Proc. Natl. Acad. Sci. U S A 97, 13913–13918. 10.1073/pnas.250478897 11095744 PMC17675

[B7] Calvert P. D. Strissel K. J. Schiesser W. E. Pugh E. N. Jr. Arshavsky V. Y. (2006). Light-driven translocation of signaling proteins in vertebrate photoreceptors. Trends Cell Biol. 16, 560–568. 10.1016/j.tcb.2006.09.001 16996267

[B8] Christiansen J. R. Kolandaivelu S. Bergo M. O. Ramamurthy V. (2011). RAS-converting enzyme 1-mediated endoproteolysis is required for trafficking of rod phosphodiesterase 6 to photoreceptor outer segments. Proc. Natl. Acad. Sci. U S A 108, 8862–8866. 10.1073/pnas.1103627108 21555557 PMC3102416

[B9] Datta P. Allamargot C. Hudson J. S. Andersen E. K. Bhattarai S. Drack A. V.. (2015). Accumulation of non-outer segment proteins in the outer segment underlies photoreceptor degeneration in Bardet-Biedl syndrome. Proc. Natl. Acad. Sci. U S A 112, E4400–E4409. 10.1073/pnas.1510111112 26216965 PMC4538681

[B10] Ding J. D. Salinas R. Y. Arshavsky V. Y. (2015). Discs of mammalian rod photoreceptors form through the membrane evagination mechanism. J. Cell Biol. 211, 495–502. 10.1083/jcb.201508093 26527746 PMC4639867

[B11] Fukada Y. Takao T. Ohguro H. Yoshizawa T. Akino T. Shimonishi Y. (1990). Farnesylated γ-subunit of photoreceptor G protein indispensable for GTP-binding. Nature 346, 658–660. 10.1038/346658a0 2385292

[B12] Gaudet R. Bohm A. Sigler P. B. (1996). Crystal structure at 2.4 Å resolution of the complex of transducin βγ and its regulator, phosducin. Cell 87, 577–588. 10.1016/s0092-8674(00)81376-8 8898209

[B13] Giessl A. Trojan P. Rausch S. Pulvermuller A. Wolfrum U. (2006). Centrins, gatekeepers for the light-dependent translocation of transducin through the photoreceptor cell connecting cilium. Vision Res. 46, 4502–4509. 10.1016/j.visres.2006.07.029 17027897

[B14] Gopalakrishna K. N. Doddapuneni K. Boyd K. K. Masuho I. Martemyanov K. A. Artemyev N. O. (2011). Interaction of transducin with uncoordinated 119 protein (UNC119): implications for the model of transducin trafficking in rod photoreceptors. J. Biol. Chem. 286, 28954–28962. 10.1074/jbc.M111.268821 21712387 PMC3190703

[B15] Higgins J. B. Casey P. J. (1994). *In vitro* processing of recombinant G protein γ subunits. Requirements for assembly of an active β γ complex. J. Biol. Chem. 269, 9067–9073. 8132644

[B16] Iñiguez-Lluhi J. A. Simon M. I. Robishaw J. D. Gilman A. G. (1992). G protein β γ subunits synthesized in Sf9 cells. Functional characterization and the significance of prenylation of γ. J. Biol. Chem. 267, 23409–23417. 1429682

[B17] Justice J. M. Murtagh J. J. Jr. Moss J. Vaughan M. (1995). Hydrophobicity and subunit interactions of rod outer segment proteins investigated using Triton X-114 phase partitioning. J. Biol. Chem. 270, 17970–17976. 10.1074/jbc.270.30.17970 7629104

[B18] Karan S. Zhang H. Li S. Frederick J. M. Baehr W. (2008). A model for transport of membrane-associated phototransduction polypeptides in rod and cone photoreceptor inner segments. Vision Res. 48, 442–452. 10.1016/j.visres.2007.08.020 17949773 PMC2262953

[B19] Kassai H. Aiba A. Nakao K. Nakamura K. Katsuki M. Xiong W. H.. (2005). Farnesylation of retinal transducin underlies its translocation during light adaptation. Neuron 47, 529–539. 10.1016/j.neuron.2005.07.025 16102536 PMC2885908

[B20] Kerov V. Rubin W. W. Natochin M. Melling N. A. Burns M. E. Artemyev N. O. (2007). N-terminal fatty acylation of transducin profoundly influences its localization and the kinetics of photoresponse in rods. J. Neurosci. 27, 10270–10277. 10.1523/JNEUROSCI.2494-07.2007 17881533 PMC6672661

[B21] Kolesnikov A. V. Rikimaru L. Hennig A. K. Lukasiewicz P. D. Fliesler S. J. Govardovskii V. I.. (2011). G-protein βγ-complex is crucial for efficient signal amplification in vision. J. Neurosci. 31, 8067–8077. 10.1523/JNEUROSCI.0174-11.2011 21632928 PMC3118088

[B22] Lai R. K. Perez-Sala D. Cañada F. J. Rando R. R. (1990). The γ subunit of transducin is farnesylated. Proc. Natl. Acad. Sci. U S A 87, 7673–7677. 10.1073/pnas.87.19.7673 2217200 PMC54810

[B23] Lambright D. G. Sondek J. Bohm A. Skiba N. P. Hamm H. E. Sigler P. B. (1996). The 2.0 A crystal structure of a heterotrimeric G protein. Nature 379, 311–319. 10.1038/379311a0 8552184

[B24] Lem J. Applebury M. L. Falk J. D. Flannery J. G. Simon M. I. (1991). Tissue-specific and developmental regulation of rod opsin chimeric genes in transgenic mice. Neuron 6, 201–210. 10.1016/0896-6273(91)90356-5 1825171

[B25] Lobanova E. S. Finkelstein S. Herrmann R. Chen Y. M. Kessler C. Michaud N. A.. (2008). Transducin γ-subunit sets expression levels of α- and β-subunits and is crucial for rod viability. J. Neurosci. 28, 3510–3520. 10.1523/JNEUROSCI.0338-08.2008 18367617 PMC2795350

[B26] Lobanova E. S. Finkelstein S. Song H. Tsang S. H. Chen C. K. Sokolov M.. (2007). Transducin translocation in rods is triggered by saturation of the GTPase-activating complex. J. Neurosci. 27, 1151–1160. 10.1523/JNEUROSCI.5010-06.2007 17267570 PMC6673185

[B27] Loew A. Ho Y. K. Blundell T. Bax B. (1998). Phosducin induces a structural change in transducin β γ. Structure 6, 1007–1019. 10.1016/S0969-2126(98)00102-6 9739091

[B28] Mangini N. J. Pepperberg D. R. (1988). Immunolocalization of 48K in rod photoreceptors. Light and ATP increase OS labeling. Invest. Ophthalmol. Vis. Sci. 29, 1221–1234. 3138199

[B29] McGinnis J. F. Matsumoto B. Whelan J. P. Cao W. (2002). Cytoskeleton participation in subcellular trafficking of signal transduction proteins in rod photoreceptor cells. J. Neurosci. Res. 67, 290–297. 10.1002/jnr.10120 11813233

[B30] Nair K. S. Hanson S. M. Mendez A. Gurevich E. V. Kennedy M. J. Shestopalov V. I.. (2005). Light-dependent redistribution of arrestin in vertebrate rods is an energy-independent process governed by protein-protein interactions. Neuron 46, 555–567. 10.1016/j.neuron.2005.03.023 15944125 PMC2752952

[B31] Najafi M. Calvert P. D. (2012). Transport and localization of signaling proteins in ciliated cells. Vision Res. 75, 11–18. 10.1016/j.visres.2012.08.006 22922002 PMC3514659

[B32] Najafi M. Maza N. A. Calvert P. D. (2012). Steric volume exclusion sets soluble protein concentrations in photoreceptor sensory cilia. Proc. Natl. Acad. Sci. U S A 109, 203–208. 10.1073/pnas.1115109109 22184246 PMC3252922

[B33] Pearring J. N. Salinas R. Y. Baker S. A. Arshavsky V. Y. (2013). Protein sorting, targeting and trafficking in photoreceptor cells. Prog. Retin. Eye Res. 36, 24–51. 10.1016/j.preteyeres.2013.03.002 23562855 PMC3759535

[B34] Philp N. J. Chang W. Long K. (1987). Light-stimulated protein movement in rod photoreceptor cells of the rat retina. FEBS Lett. 225, 127–132. 10.1016/0014-5793(87)81144-4 2826235

[B35] Pronin A. N. Gautam N. (1993). Proper processing of a G protein γ subunit depends on complex formation with a β subunit. FEBS Lett. 328, 89–93. 10.1016/0014-5793(93)80971-v 8344437

[B36] Rosenzweig D. H. Nair K. S. Wei J. Wang Q. Garwin G. Saari J. C.. (2007). Subunit dissociation and diffusion determine the subcellular localization of rod and cone transducins. J. Neurosci. 27, 5484–5494. 10.1523/JNEUROSCI.1421-07.2007 17507570 PMC2655354

[B37] Schillo S. Belusic G. Hartmann K. Franz C. Kuhl B. Brenner-Weiss G.. (2004). Targeted mutagenesis of the farnesylation site of *Drosophila* Gγe disrupts membrane association of the G protein βγ complex and affects the light sensitivity of the visual system. J. Biol. Chem. 279, 36309–36316. 10.1074/jbc.M404611200 15205461

[B38] Schou K. B. Pedersen L. B. Christensen S. T. (2015). Ins and outs of GPCR signaling in primary cilia. EMBO Rep. 16, 1099–1113. 10.15252/embr.201540530 26297609 PMC4576980

[B39] Simonds W. F. Butrynski J. E. Gautam N. Unson C. G. Spiegel A. M. (1991). G-protein β γ dimers. Membrane targeting requires subunit coexpression and intact γ C-A-A-X domain. J. Biol. Chem. 266, 5363–5366. 1706334

[B40] Slepak V. Z. Hurley J. B. (2008). Mechanism of light-induced translocation of arrestin and transducin in photoreceptors: interaction-restricted diffusion. IUBMB Life 60, 2–9. 10.1002/iub.7 18379987 PMC2717607

[B41] Sokolov M. Lyubarsky A. L. Strissel K. J. Savchenko A. B. Govardovskii V. I. Pugh E. N. Jr.. (2002). Massive light-driven translocation of transducin between the two major compartments of rod cells: a novel mechanism of light adaptation. Neuron 34, 95–106. 10.1016/s0896-6273(02)00636-0 11931744

[B42] Sokolov M. Strissel K. J. Leskov I. B. Michaud N. A. Govardovskii V. I. Arshavsky V. Y. (2004). Phosducin facilitates light-driven transducin translocation in rod photoreceptors. Evidence from the phosducin knockout mouse. J. Biol. Chem. 279, 19149–19156. 10.1074/jbc.M311058200 14973130

[B43] Steinberg R. H. Fisher S. K. Anderson D. H. (1980). Disc morphogenesis in vertebrate photoreceptors. J. Comp. Neurol. 190, 501–508. 10.1002/cne.901900307 6771304

[B44] Trojan P. Rausch S. Giessl A. Klemm C. Krause E. Pulvermuller A.. (2008). Light-dependent CK2-mediated phosphorylation of centrins regulates complex formation with visual G-protein. Biochim. Biophys. Acta 1783, 1248–1260. 10.1016/j.bbamcr.2008.01.006 18269917

[B45] Volland S. Hughes L. C. Kong C. Burgess B. L. Linberg K. A. Luna G.. (2015). Three-dimensional organization of nascent rod outer segment disk membranes. Proc. Natl. Acad. Sci. U S A 112, 14870–14875. 10.1073/pnas.1516309112 26578801 PMC4672767

[B46] Wang J. Deretic D. (2014). Molecular complexes that direct rhodopsin transport to primary cilia. Prog. Retin. Eye Res. 38, 1–19. 10.1016/j.preteyeres.2013.08.004 24135424 PMC3883129

[B47] Wedegaertner P. B. Wilson P. T. Bourne H. R. (1995). Lipid modifications of trimeric G proteins. J. Biol. Chem. 270, 503–506. 10.1074/jbc.270.2.503 7822269

[B48] Whelan J. P. McGinnis J. F. (1988). Light-dependent subcellular movement of photoreceptor proteins. J. Neurosci. Res. 20, 263–270. 10.1002/jnr.490200216 3172281

[B49] Zhang H. Constantine R. Vorobiev S. Chen Y. Seetharaman J. Huang Y. J.. (2011). UNC119 is required for G protein trafficking in sensory neurons. Nat. Neurosci. 14, 874–880. 10.1038/nn.2835 21642972 PMC3178889

[B50] Zhang H. Huang W. Zhu X. Craft C. M. Baehr W. Chen C. K. (2003). Light-dependent redistribution of visual arrestins and transducin subunits in mice with defective phototransduction. Mol. Vis. 9, 231–237. 12802257

