# Supplementary figures and images for: Farnesylation of the Transducin G Protein Gamma Subunit Is a Prerequisite for Its Ciliary Targeting in Rod Photoreceptors
Source: Front Mol Neurosci. 2018 Jan 23;11:16. doi: 10.3389/fnmol.2018.00016 (PMC5787109; doi:10.3389/fnmol.2018.00016)

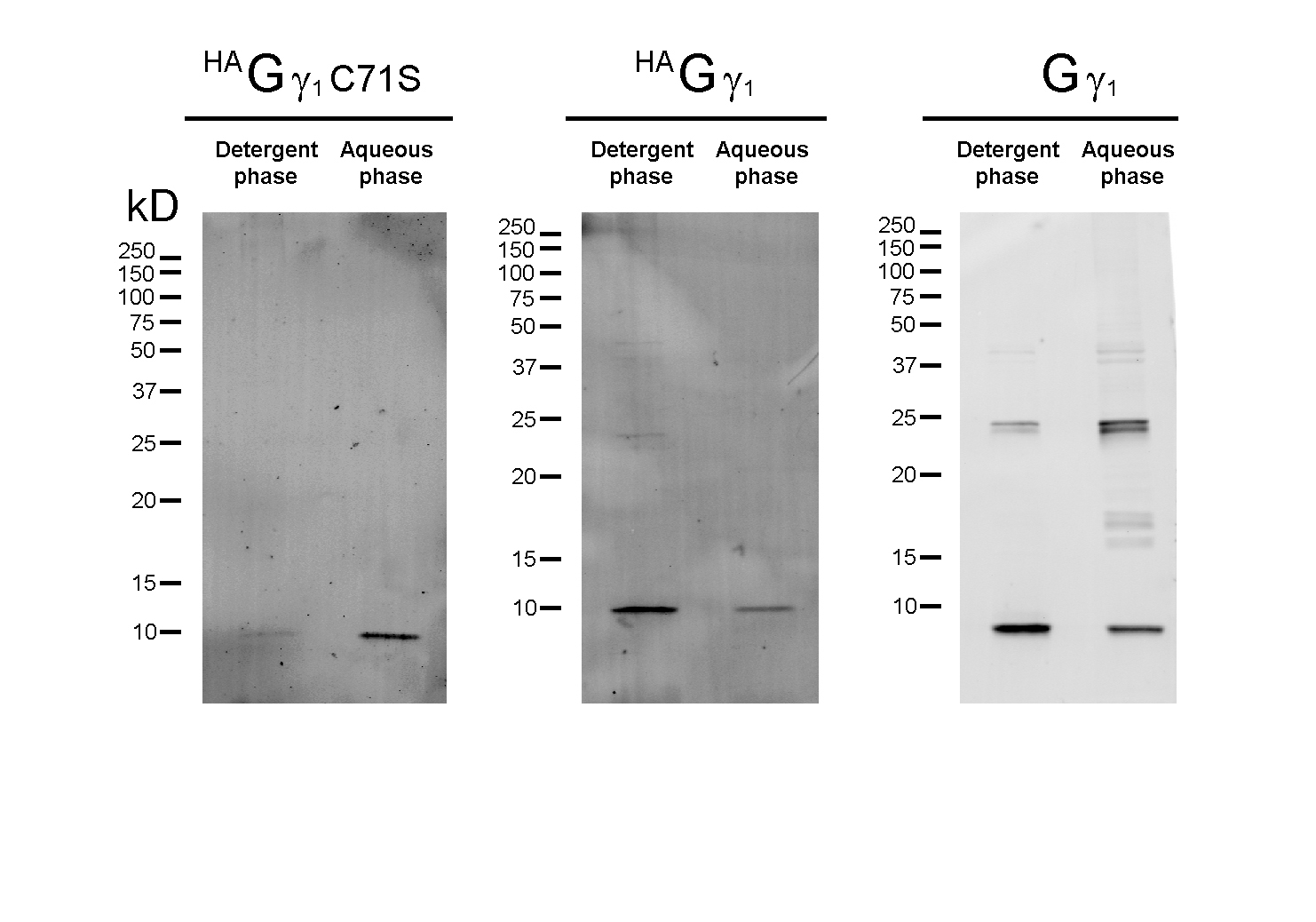

Supplement: FIGURE S1 — Full blots from in Figure 1E illustrating partitioning of HAGγ1C71S, HAGγ1, and endogenous Gγ1 between the detergent (Triton X-114) and aqueous phases. Specific bands were visualized by Western blotting with anti-HA (HAGγ1C71S, HAGγ1) and anti-Gγ1 (Gγ1). [file Image_1.jpeg]

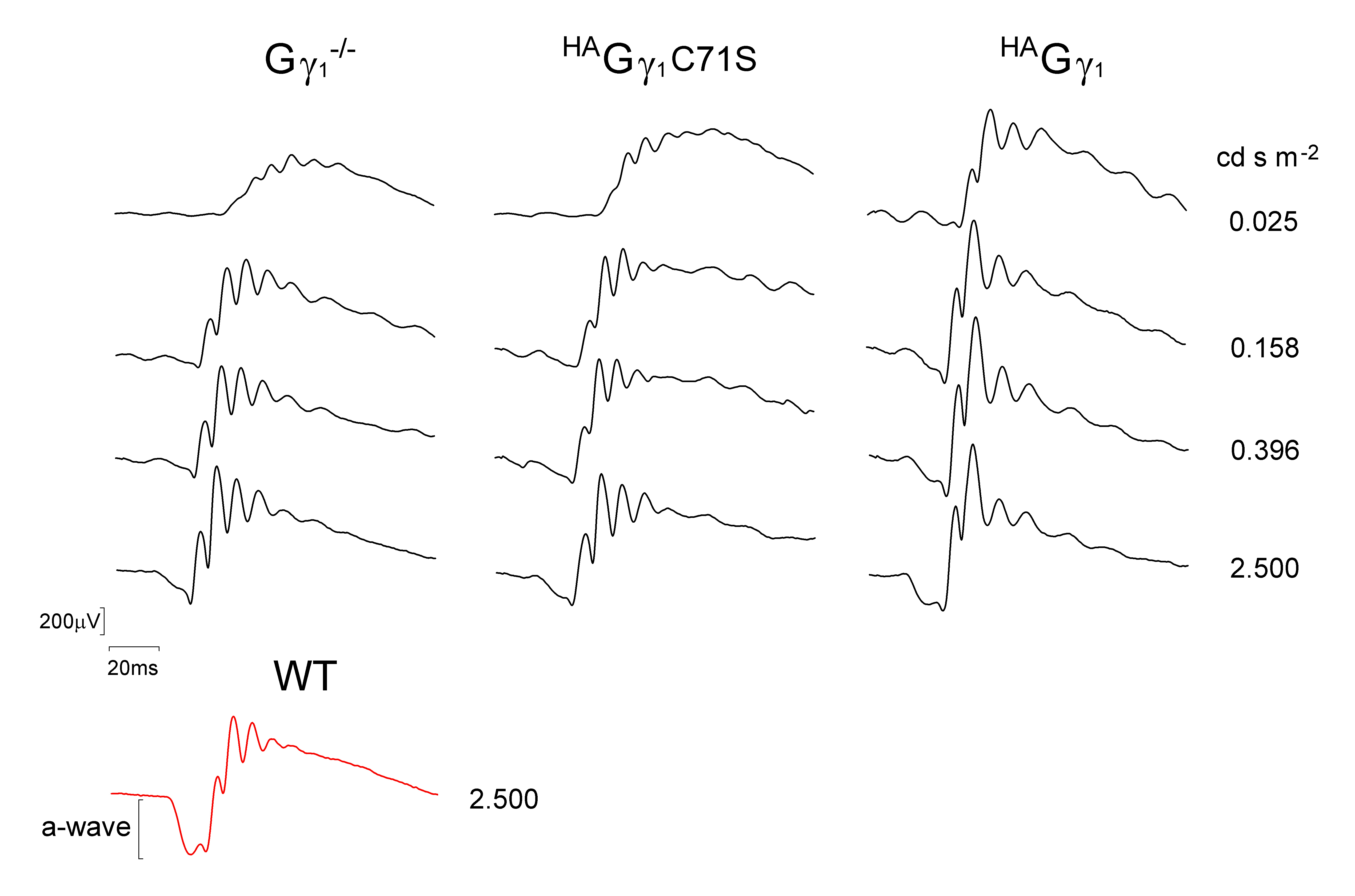

Supplement: FIGURE S2 — Representative electroretinography (ERG) recordings from Figure 2A. Visual responses were obtained using Gγ1−/−(HAGγ1−/−; Gγ1−/−), HAGγ1 (HAGγ1+/−; Gγ1−/−), and HAGγ1C71S (HAGγ1C71S+/–; Gγ1−/−) mice stimulated by flashes of indicated strength. Red trace is a typical response of a wild type (WT) mouse to a saturating 2.5 cd s m−2 flash. [file Image_2.jpeg]
